# Neuroanthropology of shamanic trance: a case study with a ritual specialist from Mexico

**DOI:** 10.3389/fpsyg.2024.1325188

**Published:** 2024-03-05

**Authors:** Hugo Toriz, Antonella Fagetti, Guadalupe Terán-Pérez, Roberto E. Mercadillo

**Affiliations:** ^1^National School of Anthropology and History, Mexico City, Mexico; ^2^Instituto de Ciencias Sociales y Humanidades, Benemérita Universidad Autónoma de Puebla, Puebla, Mexico; ^3^Sleep and Neurosciences Center, Mexico City, Mexico; ^4^Neurosciences Area, Biology of Reproduction Department, Universidad Autónoma Metropolitana, Mexico City, Mexico; ^5^CONAHCYT, Mexico City, Mexico

**Keywords:** shamanism, trance, limpia, divination, electroencephalography, consciousness, neuroanthropology

## Abstract

In Mexico, shamans are recognized for the gift of entering a deep trance that allows them to know the origin of the diseases and conflicts that afflict people. They commonly treat patients through *limpias* (cleansing) to extract negative elements sent by a witch or that were “collected” in places that harbor “evil winds.” We present a case study of an 81-year-old Mexican shaman who noticed her gift in childhood. Electroencephalographic recordings were made while the shaman performed three activities: reading cards to diagnose a patient and answer the questions he posed; *limpia* with chicken eggs, stones, and bells to absorb adverse “things”; and the incorporation trance through which the deceased is believed to occupy the shaman’s body to use it as a communication channel. Alpha activity was observed when concentrated, suggesting a hypnagogic-like state. Predominant beta and gamma oscillations were observed, suggesting a potential plastic phenomenon that modulates the assimilation of external and internal referents guiding temporal schemes for action, attention, and the integration of mnemonic, sensory, and imaginative elements. We used a neuroanthropological approach to understand shamanic trance as a biological potential of the human brain to induce non-ordinary states of consciousness linked to cultural beliefs and practices.

## 1 Introduction

For decades, anthropologists and ethnologists have used the term “shaman” interchangeably to refer to people believed to be endowed with particular characteristics, specifically the ability to access a part of reality hidden from most human beings. On behalf of the community they serve, and with the belief that they have the help of allied spirits or guardians, they enter what appears to be a deep trance or altered state of consciousness through which they experience what is perceived to be the establishment of relationships with immaterial entities to alter the order of the cosmos according to their interest or desire (Hultkrantz, 1973). As expressed by the philosopher Mircea Eliade (2013), the term “shaman” has been extended and applied to studies of religious history in several cultures, and similarities have been proposed between so-called “shamans” or “ritual specialists” throughout the world (Winkelman, 1990).

In Mexico, shamans are recognized for their ability to experience non-ordinary states of consciousness that allow them, through divination using various means (deck of cards, eggs, corn, water, copal or candles, to name a few), to discover the origin of diseases and conflicts that afflict the people who consult them. Based on auscultation or prediction, the patient is treated through a procedure called “*limpia*” (cleansing) whose function is to extract from the body the negative energies sent by a witch or that were “collected” in places that harbor “*malos aires*” (bad winds). The patient’s body can be literally swept with aromatic herbs, candles, and chicken eggs. In particular, the latter are used for diagnosis; the eggs are broken or poured into a glass of water and “read,” that is, the visible signs are interpreted, but information is also received by virtue of the communication that the shaman establishes with their guardians, a capacity that they attribute to “*el don*” (the gift), having been designated by some divinity to carry out the work of fortune tellers, healers, and spiritual guides (Fagetti, 2015).

Shamanic trances have been considered states of “concentration” during which those chosen receive messages: visual or auditory perceptions that are continually experienced in divination and healing. Practices related to trances would imply cultural adaptations, which favor the expression of the biological potential of the human brain to induce non-ordinary states of consciousness (Dobkin de Rios and Winkelman, 1989). Winkelman (2000, 2010) proposes that these trances depend on an integrative mode of consciousness, a mental state typical of shamanic practice. The author suggests that the ontological and functional bases of this state come from the functional integration of information from multiple synapses distributed in different brain regions. Because the universal principles of the healer–patient transference are implicit in therapeutic relations, Winkelman maintains that the integration of neural information allows the shamans – among other aspects of their work – to establish a deep connection with the people who require their help.

To our knowledge, there are no experimental reports on the brain function underlying shamanic trance, although some exist on states of consciousness possibly similar to this trance. Most of these reports come from electroencephalogram (EEG) recordings that indicate five waves or signals with different frequencies (Hertz) associated with differentiated physiological, cognitive, and behavioral states and processes: delta (0.5–4 Hz) associated with deep sleep; theta (4–8 Hz) with basal sleep activity; alpha (8–13 Hz) with wakefulness at quiet rest; beta (13–30 Hz) with conscious wakefulness; and gamma (>30 Hz) with conscious perception, memory, and complex thinking (Malik and Ullah, 2017; Chaddad et al., 2023). Transcendental meditation is associated with brain activity that oscillates between alpha and beta waves (Lyubimov, 1999). Hypnosis has been associated with theta and alpha frequencies, although gamma activity is particularly recurrent in people with high hypnotizability (De Pascalis and Santarcangelo, 2020; Callara et al., 2023); in Tibetan Buddhist monks, an increase in gamma frequencies has been reported during compassion meditation (Lutz et al., 2004). Other neuroimaging techniques have been used in some reports. Buddhist meditators evaluated by single-photon emission computed tomography (SPECT) showed increased blood flow in the frontal cortex, while it decreased in the parietal region, and these conditions were associated with increased self-control and sensitive-cognitive processing and a sense of inner freedom (Hankey, 2006). An interesting case is that of a woman with voluntary out-of-body experiences and whose functional magnetic resonance imaging (fMRI) indicated the activation of supplementary motor and orbitofrontal regions related to action imagery and cognitive monitoring (Smith and Messier, 2014).

Given the complexity of the shamanic trance, we propose to explore it from a neuroanthropological perspective, that is, to consider that the brain function underlying a behavior or cognitive expression not only involves the evolutionary and physiological properties of the human nervous system but also the history, dynamics, cultural beliefs, and practices that define a person and their community and that provide the nervous system with the necessary information for the person to interact with and interpret their environment (Lende and Downey, 2012a,b). To do this, we show the case of a ritual specialist or shaman with a long career. While we show some specific neurobiological and behavioral findings here, the research is part of a larger work described by the anthropologist Toriz (2018).

## 2 Method

### 2.1 Participant description

Lupita is an 81-year-old ritual specialist who lives in the state of Puebla, Mexico. According to Fagetti (2015), she was still a child – around 9 years old – when she began to have her first experiences with the shamanic gift, such as being able to “see” who was going to die, when, and under what circumstances. It was her grandmother who told her that she would perform a ritual to “open her brain,” that is, perform what is known as shamanic initiation.

According to Eliade (2013), the “ecstatic” election or first manifestations of the shamanic gift are generally followed by a period of instruction during which the neophyte is properly initiated by an ancient master. It is then when the future shaman must learn to master their mystical techniques and assimilate the religious or mythological tradition of the corresponding region and population. Lupita began to heal when her grandmother had already died; she died in Lupita’s arms at the age of 80. Like her grandmother, throughout her life, Lupita has worked as a midwife and healer. She treats her patients by reading cards and performing *limpias*. She cures conditions that traditional indigenous Mexican medicine considers “affections of the spirit” because they damage the energetic–spiritual part of the human being. They involve the loss of the spirit, such as *susto* (scare) or the penetration into the body of a pernicious energy, as in the case of “air,” when, for example, the spirit of a deceased person lodges in the body; the *mal de ojo* (evil eye) or the force that a person gives off, involuntarily, through their gaze; and damage from witchcraft or any type of curse perpetrated against someone, which causes various signs and symptoms (Fagetti, 2015).

Lupita also has the gift of contacting and interacting with deceased people to “give them light” – that is, to guide those spirits who, after dying, cannot find the way to eternal rest. Lupita says that much of her work is done under the protection of her guardians, the spirits of two people, whom she calls Huichil and Lirio, who lived in Mexico before the Spanish Conquest and who were dedicated to healing at that time (Fagetti, 2015; Toriz, 2018).

### 2.2 Assessment

Accompanied by the research team, Lupita went to the Sleep Disorders Clinic of the Metropolitan Autonomous University, Iztapalapa Unit. There, a relaxed and comfortable atmosphere was established, and the recording instruments and their use were shown and explained to her. Commonly, shamanic practices are performed in the shaman’s own environments and spaces. However, Lupita usually performs her activities in various contexts, namely, her own consulting room, her patients’ homes, or public spaces, when she is invited to an event, which is why her stay at the clinic was not experienced by her as a space outside her usual praxis.

For the EEG recording, a Cadwell Easy II device was used, with a longitudinal assembly using conventional electrodes with a gold cup, under awake conditions and following the International 10–20 System and recommendations of the International Federation of Societies of Clinical Neurophysiology, with 1 to 50 filters and 7 sensitivity, equivalent to 50 μV (San-Juan and Bermeo-Ovalle, 2023).

During the EEG, Lupita performed several tasks or activities, typical of her gift and shamanic practice, aimed at three participants who were part of the research team. The first activity (approximately 14 min) was a card reading with a Spanish deck. With this, she sought to answer three questions individually generated by the participant according to their own doubts and interests. The participant was sitting in front of a table on which Lupita placed a handkerchief and, on top of it, the deck of cards to perform the reading. After unfolding the deck and observing it carefully, she indicated to the participant her interpretation in response to each of their questions.

The second activity was a *limpia* (approximately 8 min). Lupita inherited from her grandmother a set that she covers with a red handkerchief – the contents of which she cannot reveal – and that represents the gift. With it, she covered the participant’s entire body, from head to toe, to eliminate possible damage. She “cleaned” the participant with a chicken egg and a crystal stone to see specific details about this person and give them “light” and used a small metal bell to restore their positive energy. She then ran her hands over the participant’s body. Finally, Lupita carefully observed the surface of the egg to “see” the problems that afflicted the participant and communicate them.

The third activity (approximately 14 min) consisted of an “incorporation trance.” The shaman can serve as a channel allowing the “entry” of the spirit of someone who has died. It also constitutes a shamanic technique through which an ancient healer or a Catholic saint occupies the shaman’s body and “performs” the diagnosis and *limpia*. A ritual specialist who has participated in Fagetti (2015) research explains that, when one of his guardians “arrives,” his “spirit comes out and stays above, floating.” In the case of a deceased person, this is an opportunity for them to come forward and speak to their relatives. As for the guardians of the ritual specialist, they are the ones who interact directly with the patient. Through this type of trance, Lupita sought to establish contact with a deceased man known to the third participant so that they could interact and have a dialogue, in addition to giving him peace and helping him find the light. During the trance, Lupita was sitting with her eyes closed to facilitate the link with the spirit and allow it to enter her body. Once inside, and using Lupita’s body and voice, this spirit and the participant spoke.

The three activities performed during the EEG were video-recorded and subsequently reviewed and analyzed to define three simultaneous aspects: behavioral expressions, movements, or postures; the linguistic aspects or words expressed by Lupita and the participants; and the EEG. For interpretation, the three aspects were grouped into action units or time periods defined by the meanings of each performed action. Once the action units were reviewed, the analysis integrated the “explanation” aspect or meanings defined by Lupita herself in subsequent interviews conducted in her consulting room. Lupita and the researchers watched the videos together, and based on the defined action units, Lupita provided the explanation or meanings of each of them.

**Figure 1:**
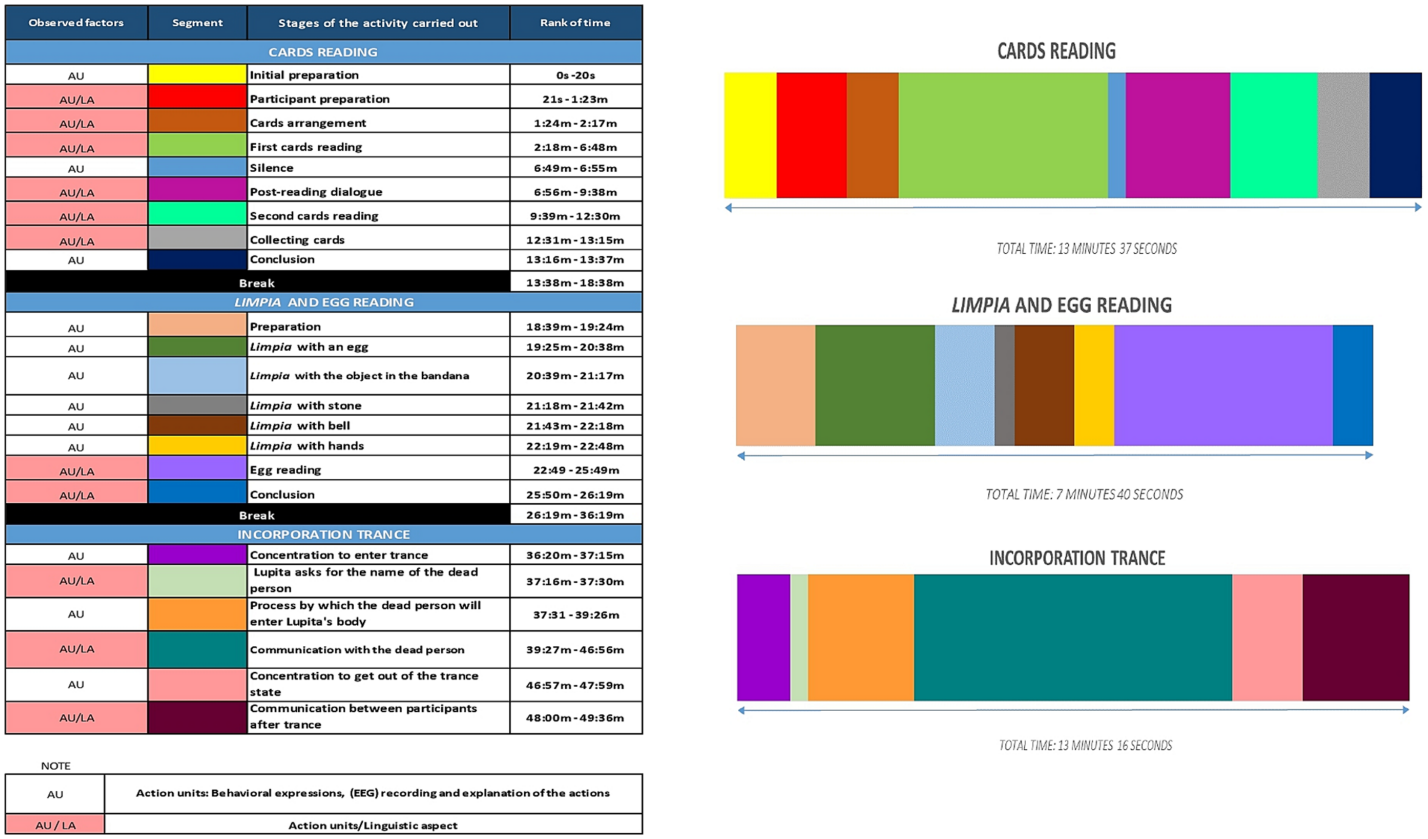
Time and sequence of behavioral, linguistic, and EEG recording for action units involving the three activities performed by the participant.

The times and sequences of the behavioral, linguistic, and EEG expressions for action units involving the three activities are shown in Figure 1.

## 3 Results

Observations and measures for each of the three activities (card reading, *limpia*, and incorporation trance) are presented in the three tables. Each table shows the different sections shaping the activity, from initial preparation to conclusion. Each section indicates the action units and the EEG time in which they were expressed. Each action unit illustrates the associated behavioral expressions, linguistic aspects, and EEG signals. In addition, it includes the explanation that Lupita gives for such actions.

### 3.1 Card reading

Lupita explains that, since her grandmother “paved the way for her,” she can read cards in a state of introspection achieved through prayers and entrustments to Catholic saints or their guardians, Huichil and Lirio (see Table 1).

**Table 1:** Behavioral expressions, linguistic aspects, electroencephalographic (EEG) recording, and explanation of the actions concerning the card reading.

Initial preparation		
Action units		
Introspection and prayer 0.01–0.20 min.	Behavioral expressions	Lupita remains silent as she holds the deck of cards in her right hand and keeps her left hand on her face, with her eyes closed.
	Linguistic aspect	-
	EEG	Beta activity and predominant 50 Hz gamma activity.
	Explanation	Lupita explains that, since her grandmother paved the way for her, she can read cards when she is in introspection and make prayers and entrustments to Catholic saints or to her pre-Hispanic protectors, Huichil and Lirio.
Participant preparation		
Action units		
Cards on the table and communication between Lupita and the participant 0.21–0.57 min.	Behavioral expressions	Lupita places the deck of cards on the table. She places her right hand on her right forearm and asks the participant to ask the questions. Lupita remains silent.
	Linguistic aspect	Lupita asks the participant to divide the cards into three groups with their right hand. She arranges the cards and asks the participant to place his right hand on them and to repeat after her: “*For you and for me… and for what I want to know*…”
	EEG	Gamma activity from 30 to 60 Hz.
	Explanation	Lupita establishes a bond of trust so that the participant can relax and begin to express their concerns. She can tell that a person has severe damage “from the moment she sees them,” and the order in which the cards appear allows her to confirm her vision.
Questions. 0.58–1.18 min.	Behavioral expressions	After the participant asks the first question, Lupita moves her head up and down indicating affirmation, places her left hand on the participant’s forearm, and then her thumb on the participant’s index finger. Lupita closes her eyes while whispering something unintelligible and directs her gaze to the participant.
	Linguistic aspect	The participant asks his questions: *Am I going to do something with music? Do I have an illness? When will I find love?*
	EEG	Beta activity in the 35–50 Hz range in the right hemisphere. Diffuse activity in the left hemisphere associated with muscular activity.
	Explanation	A relationship is established with the consultant to know where his problems or doubts reside.
Reactions after questions and before reading cards. 1.19–1.22 min.	Behavioral expressions	Lupita nods with an affirmative movement of her head (up–down).
	Linguistic aspect	Lupita says to the participant, “*couple, because you have a girlfriend*…” and asks him to raise his hand from the deck of cards.
	EEG	
	Explanation	The cards are an instrument of Lupita’s mind to exercise her gift.
Card arrangement		
Action units		
Card arrangement 1.23–1.36 min.	Behavioral expressions	Lupita picks up the cards, hits the deck on the table, and spreads them using her right hand. She asks the participant to choose a card. Lupita picks up the deck of cards again with both hands and arranges them.
	Linguistic aspect	Lupita tells the participant: “Take out a card, and put it here without seeing it,” indicating a place next to the spread cards.
	EEG	Diffuse activity associated with muscle movements alternating with gamma activity from 35 to 50 Hz.
	Explanation	The cards constitute a system made up of images, which allows the establishment of random relationships between its elements, with a wide range of permutations between them. The ability to read cards came to her suddenly, without anyone teaching her; 1 day, as she herself says, “*she decided to work, suddenly she knew the meaning of the cards and what she had to do to find a solution to the problem or doubt*.”
First card 1.37–1.44 min.	Behavioral expressions	The participant chooses a card. Lupita lifts the deck of cards with both hands and arranges the deck.
	Linguistic aspect	-
	EEG	30 Hz beta activity in the right hemisphere.
	Explanation	The cards function as a connective network of meanings about life and everything experienced in it; being a system, it is susceptible to being narrated or understood by assigning it a certain order for interpretation. The chosen card indicates the luck of the participant.
Distribution/arrangement of cards. 1.45–2.17 min.	Behavioral expressions	Lupita arranges the cards and removes the card that the participant chose from the deck. He keeps the cards in his left hand.
	Linguistic aspect	-
	EEG	Diffuse activity associated with muscle movements.
	Explanation	The distribution of the cards, that is, the way in which Lupita works and arranges them, appeared to her as part of the gift; she did not learn from anyone to read them. Her grandmother, paving the way for her, perfected this technique.
First cards reading		
Action units		
Answer to first question 2.18–3.40 min.	Behavioral expressions	Lupita remains silent, observing the cards spread on the table before telling the participant what she sees.
	Linguistic aspect	Lupita tells the participant: “*Here you are studying two things […] That is, your study of school, and your study of music; are two. But thank God that you are going to advance… in both… you are going to move forward, first of all, God… the problem is that you have friends who envy you a lot, that is, your same schoolmates…, as well who have. Now I’ll tell you how to cut it! so that you do not have a problem… because that takes away a lot of your energy… The envy thing… takes away a lot of your energy.*”
	EEG	Diffuse activity associated with muscle movements alternating with gamma activity from 30 to 60 Hz.
	Explanation	Reading cards allows Lupita to locate people’s needs and know where their problems lie.
Answer to second question 3.41–5.35 min.	Behavioral expressions	Lupita remains silent and focused. Occasionally, she places her left hand on the cards.
	Linguistic aspect	Lupita says: “*your health is a little delicate… nothing serious*”; she explains the causes are related to diet, prolonged sleeplessness, and gastritis. She recommends several remedies to improve the participant’s health and tells him that he will make two trips.
	EEG	Slowing of activity in the right hemisphere that coincides with a state of relaxation without falling asleep.
	Explanation	Reading cards allows Lupita to locate people’s needs and know where their problems lie.
Answer to third question 5–36–6.48 min.	Behavioral expressions	Lupita holds the cards in her right hand and places them on the table.
	Linguistic aspect	Lupita talks to the participant about love and tells him what she sees about his future.
	EEG	Beta activity from 20 to 30 Hz.
	Explanation	Reading cards allows Lupita to locate people’s needs and know where their problems lie.
Silence (6:50–6:55) Lupita remains silent while the participant thinks about what she is going to say. Lupita looks at his face intently.		
Post-reading dialogue (min 6:56–9:38)		
Action units		
The participant speaks 6.56–7.27 min.	Behavioral expressions	Lupita listens to the participant.
	Linguistic aspect	The participant talks about a family issue; the theme revolves around the extraordinary resemblance he bears to an uncle of his, now deceased.
	EEG	Beta activity from 20 to 30 Hz.
	Explanation	She establishes a relationship with the participant in order to know what worries or ails him.
Lupita speaks 7.28–9.18 min.	Behavioral expressions	As she begins to speak, Lupita distributes some cards on the table again, looks at them. and holds the deck of cards in her left hand.
	Linguistic aspect	Lupita talks about the resemblance between the participant and his uncle and explains the background of reincarnation.
	EEG	Diffuse activity associated with muscle movements.
	Explanation	The meaning of the cards is complicated in different ways due to the combination of the series and numbers.
The participant concludes 9.19–9.38 min.	Behavioral expressions	Lupita listens, in silence, to the participant.
	Linguistic aspect	The participant tells Lupita other things he would like to know.
	EEG	Low-frequency gamma activity from 30 to 40 Hz.
	Explanation	-
Second card reading		
Action units		
Card placement 9.38–10.28 min.	Behavioral expressions	Lupita distributes the cards again on the table and talks about the issues she “saw” previously.
	Linguistic aspect	Lupita talks about the issues she “saw” previously, and the participant asks something related to how to protect himself from envy.
	EEG	Gamma activity of 40–60 Hz.
	Explanation	Each card is a mirror and not a truth or certainty in itself, so it becomes what you see in it.
Recommendations to the participant 10.29–12.13 min.	Behavioral expressions	Lupita observes the card reading to obtain a diagnosis and listens to the participant.
	Linguistic aspect	Lupita asks the participant if he or his family are Catholic. Lupita advises him to wear a red bracelet to protect himself from envy.
	EEG	Diffuse activity associated with muscle movements.
	Explanation	Lupita interprets the arrangement of the cards taking into account their symbolism to answer the three questions posed by the participant.
Card placement 12.15–12.30 min.	Behavioral expressions	For the last time, Lupita distributes cards on the table.
	Linguistic aspect	Lupita continues telling the participant what she saw in the previous cards.
	EEG	Diffuse activity associated with muscle movements.
	Explanation	Lupita, like every healer, knows how to treat people, establishing a sensory and emotional relationship with the person to instill trust. Many times it is necessary to add more cards to expand the answer.
Collecting cards		
Actions Action units		
Last card 12.30–13.00 min.	Behavioral expressions	Lupita picks up the cards with both hands. She asks the participant to separate the card that she asked him to choose at the beginning of the session, looks at the card, and tells him his lucky number. Lupita collects the letters. The participant offers his help, and Lupita rejects it.
	Linguistic aspect	Lupita tells the participant that his lucky number is three.
	EEG	Diffuse activity associated with muscle movements alternating with beta activity.
	Explanation	For many healers, their objects are sacred and cannot be touched by anyone unless they ask and obtain permission to do so.
Wrapping/safekeeping of cards 13.00–13.15 min.	Behavioral expressions	Immediately after blessing the participant, Lupita wraps the cards in a bandana.
	Linguistic aspect	Lupita blesses the participant.
	EEG	Diffuse activity associated with muscle movements.
	Explanation	There is a syncretism between the different Catholic expressions and shamanic practices performed by Lupita.
Conclusion		
Action units		
Introspection 13.15–13.37 min.	Behavioral expressions	Lupita turns toward the camera and places her left hand on her face and eyes while keeping them closed.
	Linguistic aspect	-
	EEG	Gamma activity from 35 to 50 Hz.
	Explanation	Lupita entrusts herself to her guardian entities and thanks them for their help.

### 3.2 Limpia

Lupita prepares by asking her guardians for support and paying attention to what she is seeing (see Table 2).

**Table 2:** Behavioral expressions, linguistic aspects, electroencephalographic (EEG) recording, and explanation of the actions concerning the limpia.

	Preparation (0.01–0.45 min).
Behavioral expressions	Lupita is standing with her eyes closed. She holds something tightly wrapped in a bandana in her hands. At times, she puts a hand to her forehead. At times, Lupita appears to experience spasms, sudden movements of her head, and slight moans. She opens her eyes and kisses the object contained in the bandana, then places it on the table. Lupita takes with her right hand a spray bottle containing a solution that she brought with her and takes an egg with her left hand. She sprays the space in which she will *limpiar* (clean) the participant with the solution from the spray bottle. She points to the spot she cleaned, and the participant stands there.
Linguistic aspect	-
EEG	Alpha activity alternated with gamma traces of 35–70 Hz and with activity associated with muscle movements.
Explanation	The healer concentrates and asks for support from her protectors with the purpose of “seeing” if the participant has any serious damage.
*Limpia* with an egg (0.46–1.59 min.)	
Behavioral expressions	Lupita begins the *limpia*. She touches the participant’s forehead with the egg and then the chest, marking the shape of a cross. Then, she rubs the egg around the participant’s head and continues doing so on the arms, chest, stomach, and legs. She conducts the *limpia* in silence.
Linguistic aspect	-
EEG	Beta activity alternated with activity associated with muscle movements and with high amplitude gamma activity from 35 to 80 Hz in the right hemisphere.
Explanation	The egg “collects” the bad things that the participant may have. Unlike other healers and shamans, Lupita does not break or open the egg to read it; she performs the reading on the surface, through which it “sees” the person’s problem, solution, and possibilities.
*Limpia* with the object in the bandana (2.00–2.38 min.)	
Behavioral expressions	Lupita takes the object wrapped in the bandana and starts the *limpia*. She lightly rubs the object on the participant’s head and chest, making the shape of a cross. She continues with the arms and then with the participant’s entire body. She performs this in silence.
Linguistic aspect	-
EEG	Predominant gamma activity from 40 to 70 Hz in the right hemisphere.
Explanation	Part of Lupita’s gift remains a mystery. The object wrapped in that bandana is the “Gift”; it is what takes away the harm from her patients. No one but her can see it; she indicates that “*he has the face of Huichil*,” her protector.
*Limpia* with stone (2.39–3.03 min.)	
Behavioral expressions	Lupita places the object with the bandana on the table and takes a transparent spherical stone, inside which other blue stones can be seen. She takes the stone with both hands while surrounding the participant’s neck. Lupita closes her eyes and remains silent. Then she rubs the participant’s chest and arms and places the stone in his hands, surrounded by his own. Both remain silent and with their eyes closed for a few seconds. Lupita picks up the stone from the participant’s hands and places it on the table; with her left hand, she holds the participant’s hands.
Linguistic aspect	-
EEG	Gamma activity with interference of activity associated with muscle movement.
Explanation	Each object used in the *limpia* fulfills a specific objective. Through the stone, Lupita observes what the person needs and gives them “light.”
*Limpia* with bell (3.04–3.39 min.)	
Behavioral expressions	Lupita takes an object that looks like a small bell wrapped in a blue cloth. She rubs the participant’s hands with the object and holds them open. Then, she rubs the bell and rings it in the palms of the participant’s hands, head, neck, arms, belly, and legs. When finished, she places the bell on the table.
Linguistic aspect	-
EEG	Predominance of diffuse activity associated with movements, with gamma activity during the last seconds.
Explanation	The bell serves to restore positive energy in the participant.
*Limpia* with hands (3.40–4.09 min.)	
Behavioral expressions	Lupita takes the spray bottle with the solution she had prepared. She instructs the participant to extend his hands and sprays solution on his and her own hands. She instructs the participant to rub his hands; they both do so. Lupita places her hands on the participant’s head; they both keep their eyes closed and remain silent. Lupita runs her hands along the participant’s body: arms, chest, belly, and legs. At the end, Lupita applauds and shakes her hands.
Linguistic aspect	-
EEG	Predominance of high amplitude gamma activity from 30 to 80 Hz.
Explanation	With her hands, she transmits positive energy to the participant.
Egg reading (4.10–7.10 min.)	
Behavioral expressions	Lupita takes the egg that she had previously used with the participant. She looks at it carefully and talks about what she sees. At the end of the reading, Lupita gives the volunteer the egg to hold so that he can check the difference between a “charged” egg and a “normal” one.
Linguistic aspect	Before starting to read the egg, Lupita tells the participant to extend his hands and places the egg there. She tells the participant: “*say for you, for me, and for what I want to know*.” She also tells him to ask three questions about whatever he wants. Lupita stares at the egg in the volunteer’s hands, turns it, and observes the surface carefully. She gives answers to each of the questions.
EEG	Predominant gamma activity from 35 to 70 Hz.
Explanation	Lupita sees both what the participant needs, what he must do to heal, and, sometimes, what will happen to him. For the extraordinary to occur, it is necessary for the participant to have faith and trust that things can change, that some factor in life that distresses him can improve, and that he can be cured.
Conclusion (7.11–7.40 min.)	
Behavioral expressions	After the participant leaves the room, Lupita puts her hand to her forehead and closes her eyes. She remains silent for a few seconds.
Linguistic aspect	Lupita dismisses the participant.
EEG	Beta activity.
Explanation	Lupita comes into contact with her protective entity through prayers. When someone has a severe illness, Lupita touches her forehead, since that is where these illnesses go; her forehead changes shape, feeling curved or concave.

### 3.3 Incorporation trance

The trance is believed to allow Lupita to enter an invisible world where the dead live and she can communicate with them (see Table 3).

**Table 3:** Behavioral expressions, linguistic aspects, electroencephalographic (EEG) recording, and explanation of the actions concerning the incorporation trance.

	Concentration to enter trance (0.001–0.55 min.)
Behavioral expressions	Lupita is sitting with her eyes closed. In her hands, she holds the object wrapped in the bandana. Next to her, on the table, there is a glass of water that she had previously asked to be placed there. Lupita brings the bandana object to her forehead, sighs, and makes light moans. She rubs her hands together and continues sitting.
Linguistic aspects	-
EEG	Predominant beta activity alternating with gamma activity in a range of 25 to 50 Hz.
Explanation	Lupita acts as a medium in a trance so that the deceased person “occupies” her body. She feels dizzy, a kind of fainting, before the deceased who has been called enters her.
First stage of the trance: Lupita asks for the name of the dead person (0.56–1.10 min.)	
Behavioral expressions	Lupita remains seated with her eyes closed.
Linguistic aspects	Lupita says “*Peace be with you*…” The participant also says that phrase. Lupita asks the participant “*Who do you want to talk to?*” The participant tells her the name of the person. Lupita repeats the name in a whisper.
EEG	Initial gamma activity from 30 to 70 Hz, which then oscillates to beta in ranges from 20 to 50 Hz.
Explanation	The trance allows Lupita to enter the invisible world, where the dead live, so that she can communicate with them.
Preparation for incorporation (1.11–3.06 min.) Process by which the dead person will enter Lupita’s body	
Behavioral expressions	Lupita’s body moves as if wriggling, and she moans slightly; her movements make the chair she is sitting on creak. She keeps her eyes closed and sighs intermittently. Suddenly, she raises her head and holds on to the armrest of the chair with her left hand. She makes gestures that appear to be suffering. In her right hand, she holds the object wrapped in the bandana. She shakes violently, as if in a seizure. Lupita leans back in the chair, stirs, and suddenly straightens up, sitting almost vertically, but still shaking. Intermittently, she makes guttural sounds, a kind of moan and sigh, and grimaces that evoke pain.
Linguistic aspects	-
EEG	Gamma activity from 30 to 70 Hz that then oscillates to beta in ranges from 20 to 50 Hz.
Explanation	As Lupita enters the trance, her consciousness acquires a new perspective, a form of concentration typical of her gift that allows her to access the world of the dead.
Communication with the dead person (3.07–10.36 min.) The dead person enters Lupita’s body and communicates with the participant through it	
Behavioral expressions	Lupita remains seated with her eyes closed, while the deceased “speaks” through her. Her attitude suggests that her consciousness is not there but rather that the deceased enters her body and displaces “the ordinary Lupita.”
Linguistic aspects	The dead person speaks (through Lupita): “*Who is looking for me? What is on offer?*” The participant responds with his name. The deceased asks him why he had remembered him until that moment; he tells him that he is not feeling well and that he lacks light. The deceased lets the participant know that his ex-partner (of the deceased) was in poor health and asks him to “*take good care of him…*” “*he is going to come here soon*” (referring to the world of the dead) because he is delicate of health but has not told anyone: “*he has an incurable… illness*.” The participant asks what he can do, and the deceased groans and sighs when told that his ex-partner does not want to establish communication with anyone but that the participant must insist on the friendship he had with the deceased. The deceased asks for light “*so that I can get to my place*,” “*I suffer a lot here…I have nothing…nothing…where I’m going…I’m tired*.” The deceased also tells him: “*I left some papers… about money that was owed to me; tell him to collect it… if he can collect it*.” He also tells him the place and manner in which those papers are found. The deceased asks for water and indicates that he is very thirsty. The participant offers the glass of water to Lupita, and she takes it and drinks. The participant asks the deceased why he had died if it was not his turn yet. He responds: “*They overtook me… it wasn’t my time*” (the deceased’s expression denotes sadness). The deceased blesses the participant and asks him again to take care of his partner. He says “*goodbye*.”
EEG	Predominant gamma activity from 30 to 70 Hz alternating with diffuse activity associated with muscle movements and beta activity from 20 to 40 Hz.
Explanation	The trance that Lupita enters is the means by which a dead person can enter her body to use it as a communication channel.
Lupita’s return (10:37–11:39 min.) Concentration to get out of the trance state	
Behavioral expressions	After saying goodbye, Lupita begins to moan softly; a facial expression of sadness or pain is distinguished. She convulses and shakes her body and head. She holds the object in the bandana with both hands and kisses it; then, she crosses herself. The deceased is gone, and Lupita returns to her ordinary state of consciousness.
Linguistic aspects	-
EEG	Alpha activity of 9 Hz in the first minutes and gamma activity in a range of 30 to 60 Hz predominant in the later minutes. Then, 11 Hz alpha activity again during the last few seconds when Lupita has returned.
Explanation	Lupita must perform a particular type of concentration to come out of the trance at will. Her gift allows her to give light to the deceased when he leaves.
Communication between participants after trance (11.40–13.16 min)	
Behavioral expressions	After coming out of the trance, Lupita looks tired and as if she had been crying.
Linguistic aspects	Lupita asks if the deceased communicated: “*Did he speak?*” The participant answers yes and explains to Lupita that the deceased told him he needed light. Lupita tells him that he must light a white candle along with six white flowers and a paper with the name of the deceased written on it; when the candle burns out, he must throw away the glass, the flowers, and the paper: “*So the deceased will get light… That’s all*.”
EEG	Beta activity alternating with diffuse activity associated with muscle movements.
Explanation	Thanks to her gift, Lupita can establish contact with the dead for various purposes, but the main one is to give them “light” so that they can find the path to peace. Lupita is not aware of her speech or movements while the deceased is in her body.

## 4 Discussion

During the card reading, the EEG predominantly showed beta activity when Lupita was listening to the participant; beta signals also occur during transcendental meditation (Lyubimov, 1999) and perhaps involve phonological and semantic processes (Spironelli et al., 2013). In Lupita’s case, beta activity may have been necessary for her to understand the participants and their questions about the cards. Gamma activity was also recorded with frequencies up to 60 Hz, suggesting complex cognitive processes involving attention and memory for both sensory and non-sensory aspects (Jensen et al., 2007). In fine EEG studies, it has been suggested that this rapid activity originates in the hippocampus and is the result of interactions of the hippocampus with the neocortex, which may explain memory consolidation and explicit learning processes (Pedrosa et al., 2022), necessary to integrate previously known and recently learned elements shaping new connections. The cognitive complexity involved in card reading perhaps corresponds to observing the arrangement of the cards on the table, a moment in which Lupita “listens,” as she explained, to her guardians, who suggest the answers to the questions asked by the consultant.

Regarding the *limpia*, particularly during the initial preparation, alpha activity involving resting and relaxed but attentive states of wakefulness was observed (Sanei and Chambers, 2007). Some authors have suggested that alpha activity is the first EEG element affected by preparation for a non-ordinary state of consciousness – for example, in hypnosis or in transcendental meditation that elicits a sensation of completeness in practitioners with several years of experience (De Pascalis and Santarcangelo, 2020; Callara et al., 2023). This alpha state would suggest that practice and training configure a state of consciousness similar to the transition to stage 1 sleep, with certain similarly hypnagogic effects (Lyubimov, 1999; Hankey, 2006). In Lupita, these effects can be understood within the framework of the body movements and postures that she performs sitting and that are meant by her as a form of concentration and request for support from her guardians [Huichil and Lirio], as well as being able to “see” if the participant has some serious damage. In contrast, during the *limpia* process itself, after preparation, the EEG showed predominantly gamma activity. This could represent a particular sensitivity of Lupita to evoke and sustain such brain function, similar to people with high hypnotizability (De Pascalis and Santarcangelo, 2020; Callara et al., 2023). It could also represent complex cognitive functions that, in the case of Lupita, include an understanding toward the patient and the intention for well-being translated into the use of her gift and in practices such as collecting, with the egg, the “bad things” that the patient may have, observing what they needs in the stone, or reestablishing their positive energy with the bells and hands. It is notable that gamma activity has been observed in Buddhist monks when practicing compassion meditation, which, similarly to Lupita’s practices, involves an evident intention and disposition of well-being toward others (Lutz et al., 2004). At the end of the *limpia*, after reading the egg and saying goodbye to the participant, the EEG recorded 20 Hz beta activity, typical for normal active wakefulness. The EEG during the *limpia* could represent a particular state of concentration that Lupita enters at will. The alternation of gamma and beta frequencies could signify a plastic phenomenon that modulates, through excitatory and inhibitory processes, conscious attention toward external body referents and those inner and abstract ones (Bibbig et al., 2002).

Beta and gamma alternation was also observed during the “incorporation trance,” although predominantly high-frequency gamma activity (up to 80 Hz) was recorded, particularly at the time when Lupita was communicating with the deceased. In Mexican shamanic practices, incorporation implies that the shaman’s spirit “goes out and remains above, floating” (Fagetti, 2015), a similar description to that reported by a woman with the ability to leave her body at will (Smith and Messier, 2014). However, unlike this last case in which the out-of-body experience was associated with brain functions involved in motor monitoring and imagination, Lupita (and shamans in general) is not aware of her body or the interaction she maintains with the participant; it is the spirit of the deceased, his consciousness, that speaks through Lupita. In the end, when saying goodbye and once the deceased man left Lupita, low-frequency alpha activity was recorded, which may be associated with the resting wakefulness that precedes sleep, as has been observed in meditators (Cahn and Polich, 2006) and that coincides with Lupita’s exhaustion at the end of this activity.

Lupita’s EEG showed predominantly gamma activity alternating with beta activity during the three performed actions. These oscillations are consistent with what was observed in Tibetan Buddhist monks while practicing compassion meditation (Lutz et al., 2004) and in states of hypnosis in people with high hypnotizability (De Pascalis and Santarcangelo, 2020; Callara et al., 2023). Such oscillation perhaps represents a conscious and equanimous preparation elicited by neuronal assembly and plastic activity in the cortex, indicating temporal schemes of action, rapid and flexible thinking, attention, and integration of mnemonic, sensory, and imaginative elements (Fries et al., 2007). Moreover, it may involve training in the case of meditation and induced suggestion in the case of hypnosis, and both circumstances may be present in Lupita’s shamanic practice. However, unlike contemplative practices and hypnosis, shamanism implies a gift understood from worldviews and conceptions that make the shaman a vehicle. Thus, said brain function can refer to the integrative mode of consciousness or mental state typical of shamanic activity, as suggested by Winkelman (2010). Following Dobkin de Rios and Winkelman (1989), this mental state would imply a biological potentiality adapted to Lupita’s culture and from which the meanings of her practices, such as “seeing” what is bad about the patient, communicating with her guardians, or speaking with a deceased person, have a psychophysiological correspondence. Furthermore, this brain function implies training and practice, as has been suggested for Buddhist monks (Cahn and Polich, 2006), that, in the case of Lupita, although attributable to her gift, are also guided by her guardians, having been trained by her grandmother since she was a child and having constantly practiced since then.

Our work proposes a neuroanthropological approach to understanding shamanic trance. We understand that pure psychophysiological or anthropological analysis is insufficient and that dual interpretations are necessary to comprehend human behavior manifested in specific spaces (Pritzker et al., 2023). Furthermore, as proposed in the case of Candomblé, in Brazil (Seligman, 2010), we understand that the healer (or shaman) represents a “self” that crosses mind–body interactions and that cultural practices can influence these interactions within healing dynamics. Therefore, as proposed by Lende et al. (2021), we realized an interdisciplinary methodological triangulation to link observations made in the field with evaluations from an experimental laboratory. In this way, a conceptual triangulation between anthropology, psychology, and neuroscience is possible to jointly interpret the results from three disciplinary approaches that denote the mental, physical, and social aspects of the phenomenon. To complement our interpretation, we included what Lende et al. (2021) call “Say” and “Do,” that is, Say, which corresponds to the participant’s explanations, in their own words, about how they interpret what happens and why, and Do, which corresponds to the practices performed in specific contexts.

Our proposal is an exploratory case study with several limitations: EEG interpretations require additional analyses to define brain topography or use control situations and larger samples to contrast our findings. However, although various shamanic practices share common elements, they also take into account the particularity of each shaman’s history, community, and beliefs. A future neuroanthropological path could encompass more and varied elements of the shaman’s own culture – for example, the participant or patient being a member of the shaman’s community with a greater affinity of traditions and beliefs. Currently, it would be possible to adopt ambulatory methods, such as portable EEG instruments, to perform recordings in the shaman’s own spaces and thus achieve more ecological information (Pritzker et al., 2023). Likewise, given that the shaman’s work fundamentally involves healing in the shaman–patient relationship, the possible electroencephalographic synchronization between both and during the process could be investigated and integrated with the people’s narratives of healing.

## 5 Patient perspective

Throughout her more than 20 years of research on shamanism in Mexico, Antonella Fagetti and Lupita have formed a bond of trust, thanks to which, after having our objectives and procedures explained to her, she agreed to participate in this research and provided her informed consent. Lupita has always shown interest in openly collaborating in any exercise that, as mentioned, can contribute to the clarification of the mental processes involved in shamanic praxis. The three participants in the activities performed by Lupita were members of the research team and agreed to voluntarily participate because of their interest in answering their own questions. The research followed the guidelines proposed by the Declaration of Helsinki and was approved by the ethics committee of the Universidad Autónoma Metropolitana, Unidad Iztapalapa.

## Data availability statement

The raw data supporting the conclusions of this article will be made available by the authors, without undue reservation.

## Ethics statement

The studies involving humans were approved by Ethics Commission, Universidad Autónoma Metropolitana, Unidad Iztapalapa. The studies were conducted in accordance with the local legislation and institutional requirements. The participants provided their written informed consent to participate in this study. Written informed consent was obtained from the individual(s) for the publication of any potentially identifiable images or data included in this article.

## Author contributions

HT: Conceptualization, Data curation, Investigation, Methodology, Writing – original draft, Formal analysis, Writing – review & editing. AF: Conceptualization, Formal analysis, Investigation, Methodology, Writing – original draft, Funding acquisition, Supervision, Writing – review & editing. GT-P: Formal analysis, Funding acquisition, Methodology, Writing – original draft, Resources, Writing – review & editing. RM: Formal analysis, Funding acquisition, Methodology, Resources, Writing – review & editing, Conceptualization, Data curation, Investigation, Project administration, Supervision, Writing – original draft.
